# Descriptions of four new dextral land snails of the genus *Camaena* (Gastropoda, Eupulmonata, Camaenidae) from south China

**DOI:** 10.3897/zookeys.996.54187

**Published:** 2020-11-24

**Authors:** Pei Wang, Mei-Ling Hu, Jun-Hong Lin, Hai-Fang Yang, Xiao-Jing Li, Wei-Chuan Zhou

**Affiliations:** 1 Key Laboratory of Molluscan Quarantine and Identification of GACC, Fuzhou Customs District, Fujian 350001, China Key Laboratory of Molluscan Quarantine and Identification of GACC, Fuzhou Customs District Fuzhou China; 2 College of Plant Protection, Fujian Agriculture and Forestry University, Fuzhou, Fujian 350002, China College of Plant Protection, Fujian Agriculture and Forestry University Fuzhou China; 3 National Wetland Museum of China, Hangzhou, Zhejiang, 310013, China National Wetland Museum of China Hangzhou China

**Keywords:** Anatomy, *Camaena*, molecular biology, shell morphology, terrestrial snail

## Abstract

In this study, four new dextral camaenid from China are reported, based on shell morphology, reproductive system anatomy, and molecular phylogenetic analyses: *Camaenafuningensis* Zhou, Wang & Lin, **sp. nov.**, *Camaenagaolongensis* Zhou, Wang & Lin, **sp. nov.**, *Camaenamaguanensis* Zhou, Wang & Hu, **sp. nov.**, and *Camaenayulinensis* Zhou, Wang & Hu, **sp. nov.** Detailed descriptions of the morphological characteristics including shells and genitalia, DNA sequences, and living environments of the four new species are provided, with further comparisons with congeners.

## Introduction

The genus *Camaena* was established by Albers (1850). It is the speciose type genus in the family Camaenidae, with the type species *Helixcicatricosa* Müller, 1774. The species in this genus are mainly distributed throughout southern China, Indochina, and beyond in Southeast Asia, and most are locally endemic (Pilsbry 1894; Zilch 1959–1960, 1964; Richardson 1985; Chen and Gao 1987; Ding et al. 2016; Inkhavilay et al. 2019). The genus was divided into five subgenera (*Camaena* Albers, 1850, *Camaenella* Pilsbry, 1893, *Pseudobba* Moellendorff, 1891, *Pancala* Kuroda & Habe, 1949, *Miyakoia* Minato, 1980) on the basis of classifications by Pilsbry (1894), Kuroda and Habe (1949), Zilch (1959–1960), and Vaught (1989). A recent molecular phylogeny (Hoso et al. 2010) and anatomical study (Hwang 2012) suggested that *Pancala* and *Miyakoia* should be synonyms of the confamilial genus *Satsuma*.

There are 24 species of the genus distributed in southern China belonging to two subgenera, *Camaena* and *Camaenella*. Twenty-three species belong to *Camaena* (Yen 1939; Chen and Zhang 1999; Schileyko 2003; Ai et al. 2016; Ding et al. 2016), and only one species is in *Camaenella* (Pilsbry 1894; Yen 1939; Chen and Zhang 1999). The subgenusCamaenella was treated as a synonym of *Camaena* or as a genus in its own right by some scholars (Chen and Gao 1987; Chen and Zhang 1999). In this article, *Camaenella* will be considered as a valid subgenus.

*Camaena* species are divided into a sinistral group and a dextral one. They are usually characterized by a moderately solid shell with scar-like protrusions or malleations, 4.5–5.5 slightly convex whorls, a brown or yellow surface with red or puce spiral bands, and reflexed aperture margins (Schileyko 2003; Ai et al. 2016). The classification of *Camaena* has mainly relied on the shell features. Anatomical and molecular studies of *Camaena* are rare, except for the sinistral and the newly described species (Chen and Zhang 1999; Ai et al. 2016; Ding et al. 2016; Páll-Gergely et al. 2016; Wu et al. 2019). Historically, the classification of this genus is rather confused. For the sinistral group, the taxonomic status has always been controversial, and scientific names have been revised repeatedly. Ding et al. (2016) revised *C.cicatricosa* as four species, *C.cicatricosa*, *C.inflata* (Möllendorff, 1885), *C.obtecta* (Fischer, 1898), and *C.connectens* (Dautzenberg & Fischer, 1906), and described one new species *C.poyuensis* Zhou, Wang & Ding, 2016 using morphological and molecular studies. In the same year, Ai et al. (2016) described two new species *C.lingyunensis* Zhou & Lin, 2016 and *C.detianensis* Zhou & Lin, 2016 according to shell morphology, reproductive system and molecular biology. Thus, the sinistral *Camaena* group contains 12 species or subspecies to date (Schileyko 2003; Ai et al. 2016; Ding et al. 2016). The dextral group can be divided into three informal subgeneric groups according to the morphological characteristics of the shell, especially the shape and location of the carina.

1. Group I possesses an acute and moderate carina on the body whorl. This group could be further divided into two categories by shell height i.e., a relatively low and flat spire, which includes C. longsonensis (Morlet, 1891), C. jinpingensis Chen, Zhang & Li, 1990 and C. vorvonga (Bavay & Dautzenberg, 1900); a relatively high spire, e.g., C. vayssierei (Bavay & Dautzenberg, 1909).
2. Group II possesses a blunt carina, which is placed on the higher or middle parts of the body whorl, such as C. vulpis (Gredler, 1887), C. leonhardti (Möllendorff, 1888), and C. choboensis (Mabille, 1889).
3. Group III possesses a smooth periphery, e.g., C. hainanensis (Adams, 1870) and C. xanthoderma (Möllendorff, 1882).

In this study, the authors have examined many specimens collected in Guangxi and Yunnan in southern China between 2013 and 2015, and discovered four new dextral species on the basis of morphological, anatomical, and molecular evidence, and living environments.

## Materials and methods

**Figure 1.**
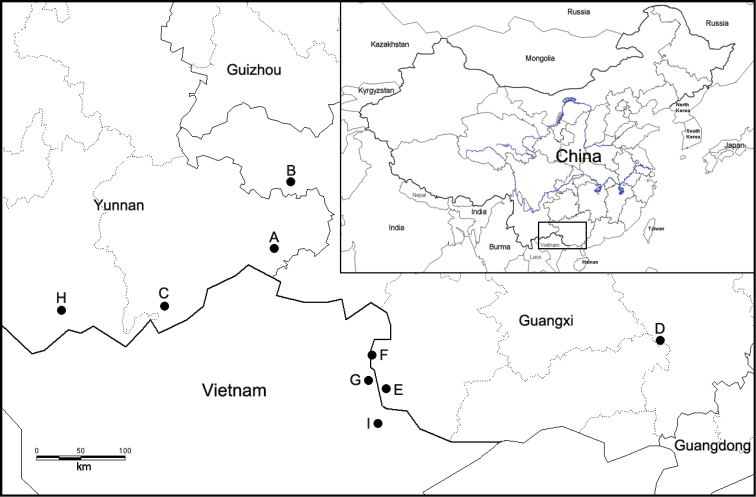
Map of locations of *Camaena* species. *C.funingensis* sp. nov. **A** Laolida, Funing, Wenshan, Yunnan, China. *C.gaolongensis* sp. nov. **B** Dayao, Gaolong, Tianlin, Guangxi, China. *C.maguanensis* sp. nov. **C** Huazhige, Maguan, Wenshan, Yunnan, China. *C.yulinensis* sp. nov. **D** Longquan cave, Yulin, Guangxi, China. *C.vorvonga***E** Pingxiang, Guangxi, China **F** Longzhou, Guangxi, China **G** That-khe, Vietnam (Type locality). *C.jinpingensis***H** Jinping, Yunnan, China. *C.longsonensis***I** Lang-Son, Vietnam.

Specimens were collected by the authors from several sites in China (Fig. 1). The longitude and latitude were recorded using a GPS. The map was established by MapInfo Professional 15.0. The live adults were drowned in water for 12–24 hours, and then killed in hot water. Soft bodies were preserved in 95% ethanol and stored at -20 °C. Empty shells were cleaned and preserved at room temperature in the Key Laboratory of Molluscan Quarantine and Identification of Fuzhou Customs District, Fujian, China (**GACC**).

Shells were measured to 0.1 mm using electronic calipers. Standard shell parameters were taken following Dillon (1984). All adult specimens of each species were measured. Live sexually mature specimens were dissected for the examination of reproductive system under a dissecting microscope (ZEISS Stemi 2000). Terminology for reproductive system follows Gómez (2001). The basal direction starts from the reproductive opening while that of verge starts from the epiphallus following Hwang et al. (2018).

Approximately 30 mg of the foot muscle was used for DNA extraction. The foot muscle was bathed in sterile water for 3–6 hours to remove residual alcohol. Genomic DNA was isolated using Qiagen DNeasy Blood & Tissue kit (Qiagen, Beijing), examined by agarose gel electrophoresis and ultra-micro spectrophotometer (Implen NP80, Germany), then stored at -20 °C for further use. The partial mitochondrial cytochrome c oxidase subunit 1 (COI) was amplified by PCR using apt primer pairs, reaction system, and amplification condition listed in Table 1. The PCR products were analyzed using 1.2% agarose gel electrophoresis.

**Table 1.** Primer pairs and PCR conditions used in the analyses of the COI gene of *Camaena*.

Gene	COI
Primer pairs (5’-3’)	LCO:GGTCAACAAATCATAAAGATATTGG
	HCO:TAAACTTCAGGGTGACCAAAAAATCA
Reaction systems	25 µl Taq PCR MasterMix × 2; 1 µl each primer; 2 µl DNA; 16 µl ddH_2_O
Cycling conditions	94 °C: 30 s; 94 °C: 10 s, 45 °C: 50 s, 72 °C: 1 min, 40 cycles; 72 °C: 10 min.
Reference	Folmer et al. 1994

After sequencing, raw sequences were proof-read on chromatograms and aligned into contigs using BioEdit 7.2 (Hall 1999). Sequence alignments were generated using ClustalW implemented in MEGA6 (Tamura et al. 2013). A total of 35 sequences were used in this study, 23 sequences of which were newly generated and deposited in GenBank (Table 2), and the remainder referenced in Wu et al. (2008), Ding et al. (2016), Ai et al. (2016), and Hu et al. (2019). Pairwise *p*-distances between taxa were calculated using MEGA6 (Tamura et al. 2013) and were compared with the currently known intra and inter- specific differentiation values (p-distances) of Camaenidae (Criscione and Köhler 2014; Ai et al. 2016; Ding et al. 2016). Neighbor-Joining and Minimum-Evolution analyses based on COI sequences were performed using MEGA6 (Tamura et al. 2013). *Amphidromusatricallosus* (Gould, 1843) (Camaenidae) was used as outgroup. The node support values were assessed by bootstrap resampling using 1000 replicates (Felsenstein 1985).

**Table 2.** Sampling GenBank accession numbers used in phylogenetic analysis.

Species	COI accession numbers	References
*Camaenafuningensis* sp. nov.	MT449465, MT449466, MT449467	Present study
*Camaenagaolongensis* sp. nov.	MT449468, MT449469, MT449470	Present study
*Camaenamaguanensis* sp. nov.	MT449471, MT449472	Present study
*Camaenayulinensis* sp. nov.	MT449473, MT449474, MT449475	Present study
*Camaenavorvonga*	MT984239	Present study
*Camaenaxanthoderma*	MT984235	Present study
*Camaenaxanthodermapolyzona*	MT984236	Present study
*Camaenahainanensis*	MT984234	Present study
*Camaenachoboensis*	MT984240	Present study
*Camaenagabriellae*	MT984241	Present study
*Camaenagabriellaeplatytaenia*	MT984242	Present study
*Camaenalongsonensis*	EF057379	Wu et al. 2008
*Camaenajinpingensis*	KU586503	Ding et al. 2016
*Camaenamenglunensis*	KU586506	Ding et al. 2016
*Camaenainflata*	KU586524	Ding et al. 2016
*Camaenaobtecta*	KU055610	Ding et al. 2016
*Camaenahahni*	KX621263	Ai et al. 2016
*Camaenaconnectens*	KU586518	Ding et al. 2016
*Camaenapoyuensis*	KU061273	Ding et al. 2016
*Camaenalingyunensis*	KX345077	Ai et al. 2016
*Camaenacicatricosa*	KU061276	Ding et al. 2016
*Camaenadetianensis*	KX345074	Ai et al. 2016
*Camaenellaplatyodon*	MH362759	Hu et al. 2019
*Camaenaleonhardti*	MT984237	Present study
*Camaenavulpis*	MT984238	Present study
*Camaenahemiclista*	MT984243	Present study
*Camaenahaematozona*	MT984244	Present study
*Amphidromusatricallosus*	MT984245	Present study

Abbreviations used in this work:

**AG** albumen gland;

**AH** aperture height;

**AW** aperture width;

**BC** bursa copulatrix;

**COI** cytochrome c oxidase subunit 1gene;

**E** epiphallus;

**F** flagellum;

**FJIQBC** Original Fujian Entry-Exit Inspection & Quarantine Bureau, Fuzhou, Fujian, China;

**GACC** General Administration of Customs, People’s Republic of China;

**HD** hermaphroditic duct;

**ME** Minimum-Evolution;

**MNHN** Muséum national d’Histoire naturelle, Paris, France;

**NJ** Neighbor-Joining;

**O** oviduct;

**P** penis;

**PBC** pedunculus of bursa copulatrix;

**PR** penis retractor muscle;

**SH** shell height;

**SW** shell width;

**V** verge;

**Va** vagina;

**VD** vas deferens.

## Results

### Molecular analysis

In this study, a total of 35 sequences of COI from 28 species were used, including eleven sequences from *C.funingensis* sp. nov., *C.gaolongensis* sp. nov., *C.maguanensis* sp. nov., and *C.yulinensis* sp. nov., 8 sequences from sinistral *Camaena* (*C.cicatricosa*, *C.obtecta*, *C.inflata*, *C.connectens*, *C.hahni*, *C.detianensis*, *C.lingyunensis*, *C.poyuensis*), 16 sequences from dextral *Camaena* and one outgroup (*A.atricallosus* Family Camaenidae) listed in Table 2.

Inter and intra-specific *P*-distances from COI gene of seven species were calculated and are listed in Table 3. According to the results of the target gene COI, the *p*-distances between *C.funingensis* sp. nov., *C.gaolongensis* sp. nov., *C.maguanensis* sp. nov., and *C.yulinensis* sp. nov. and other dextral *Camaena* were 0.068–0.200, 0.075–0.203, 0.068–0.198 and 0.092–0.202 respectively.

**Table 3.** Inter and intra-specific *P*-distances of the COI sequences on dextral *Camaena* species.

Sampling	P-distances	
	Within	Between
*Camaenafuningensis* sp. nov.	0.000	0.068–0.200
*Camaenagaolongensis* sp. nov.	0.000	0.075–0.203
*Camaenamaguanensis* sp. nov.	0.000	0.068–0.198
*Camaenayulinensis* sp. nov.	0.000–0.002	0.092–0.202
*Camaenavorvonga*	0.000–0.002	0.089–0.209
*Camaenajinpingensis*	0.000–0.002	0.196–0.209
*Camaenalongsonensis*	0.000	0.153–0.211

**Figure 2.**
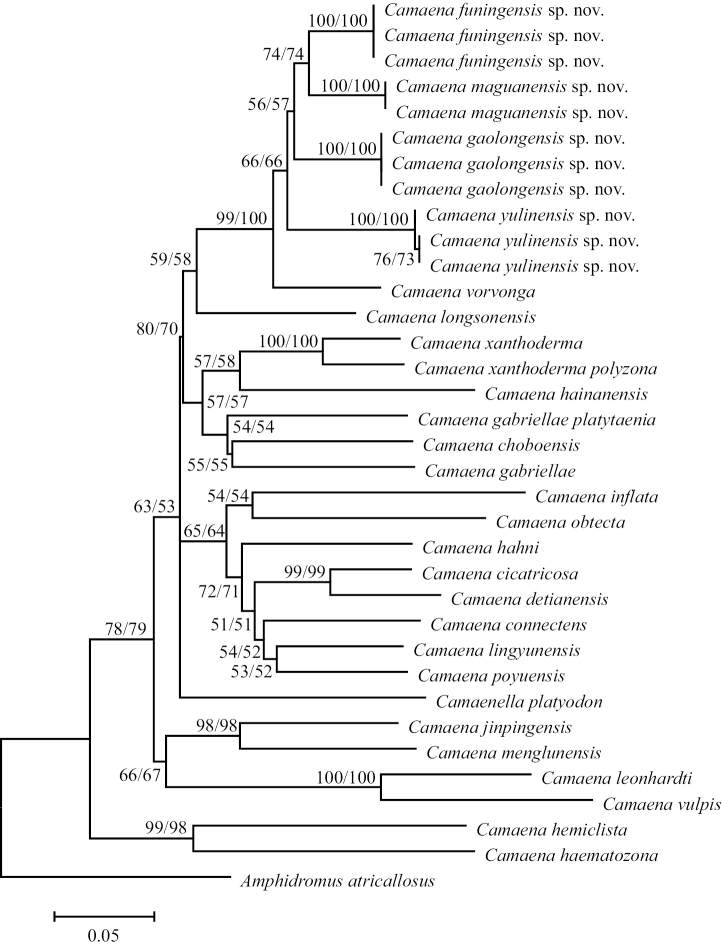
Neighbor-Joining and Minimum-Evolution trees based on analysis of the COI sequences. Numbers beside nodes indicate bootstrapping support (%) for the main clades, based on 1000 replicates.

For phylogenetic analysis, results showed that Neighbor-Joining and Minimum-Evolution trees had mostly the same topological structure (Fig. 2), and indicated that phylogenic analyses were relatively correct and reliable. The bootstrap support of each species exceeds 50%. The sinistral camaenids were clearly clustered together. The four dextral new species have the closest phylogenetic relationship to each other and are sister species with *C.vorvonga*. From the tree structure, branch length and comparison of the known species, the phylogenetic trees supported *C.funingensis* sp. nov., *C.gaolongensis* sp. nov., *C.maguanensis* sp. nov., and *C.yulinensis* sp. nov. as new species. Moreover, the four new species all had a closer genetic relationship with each other than with any other *Camaena* species studied here.

### Systematics

#### Camaenidae Pilsbry, 1895

##### Camaena

Taxon classificationAnimaliaCamaenidae

Albers, 1850

B46FE9CF-26A0-5F3E-B2D0-45B1A809E308

###### Type species.

*Helixcicatricosa* Müller, 1774, subsequent designation by Martens 1860.

##### Camaena funingensis

Taxon classificationAnimaliaCamaenidae

Zhou, Wang & Lin sp. nov.

8B931CAC-5AEE-5726-92BB-CB4DE6A19AF9

http://zoobank.org/E94E735E-BAD1-4D8C-AC91-5D50DF90AFE5

**Figure 3.**
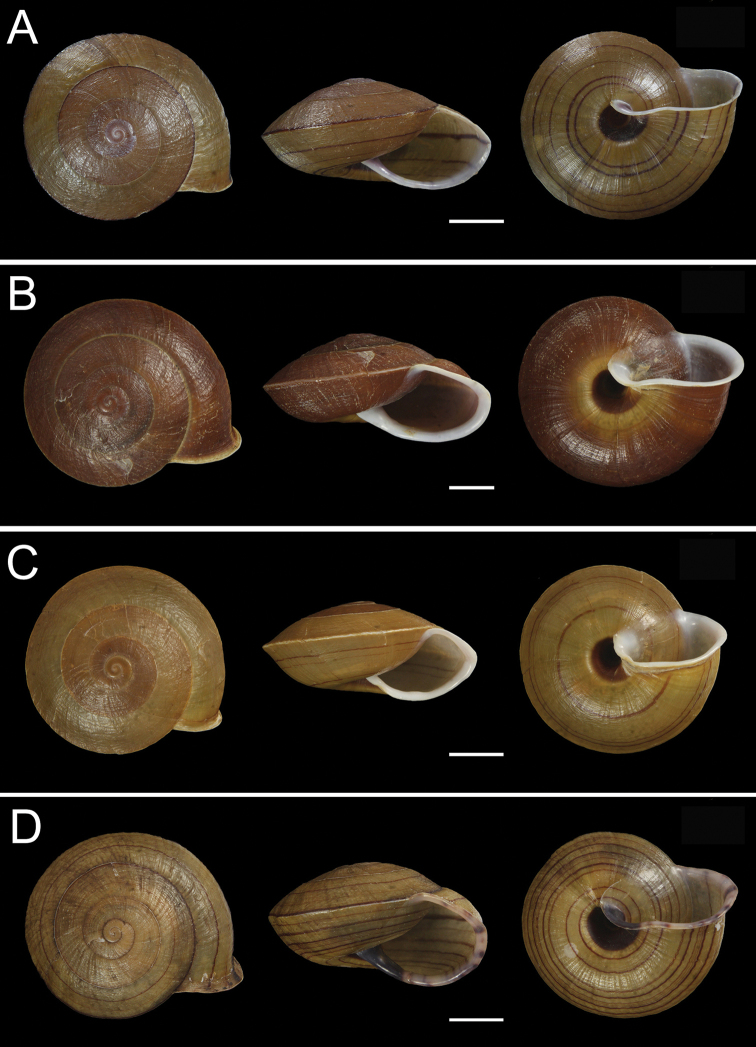
Photographs of the four new species **A***Camaenafuningensis* sp. nov. (holotype, FJIQBC 19340, Laolida, Funing, Yunnan, China) **B***Camaenagaolongensis* sp. nov. (holotype, FJIQBC 19353, Dayao, Gaolong, Guangxi, China) **C***Camaenamaguanensis* sp. nov. (FJIQBC 19405, Huazhige, Maguan, Yunnan, China) **D***Camaenayulinensis* sp. nov. (FJIQBC 19460, Longquan cave, Yulin, Guangxi, China). Scale bars: 10 mm.

**Figure 4.**
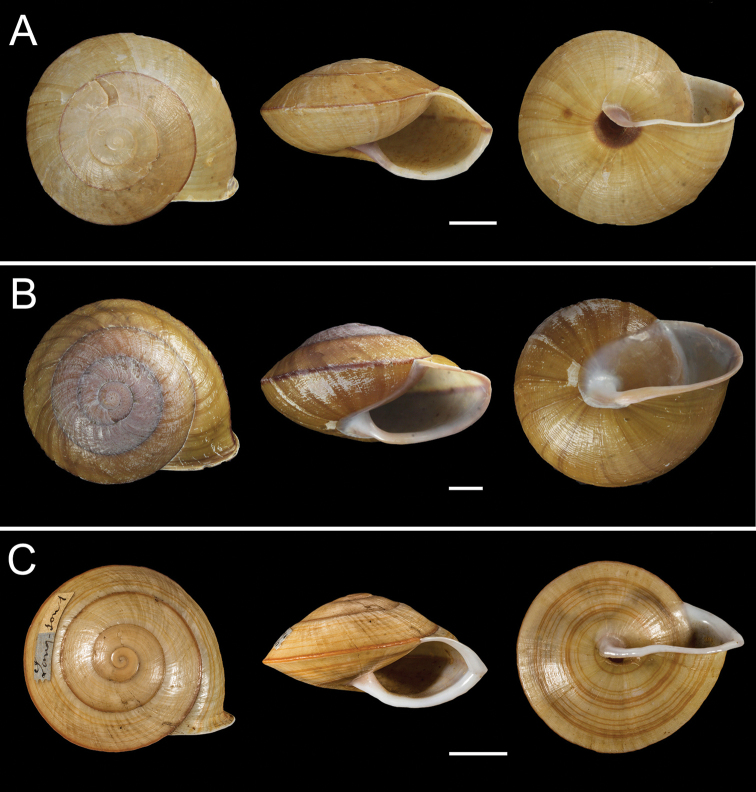
Photographs of three camaenids **A***Camaenavorvonga* (Pingxiang, Guangxi, China) **B***Camaenajinpingensis* (Jinping, Yunnan, China) **C***Camaenalongsonensis* (Lang-Son, Vietnam). Scale bars: 10 mm.

**Figure 5.**
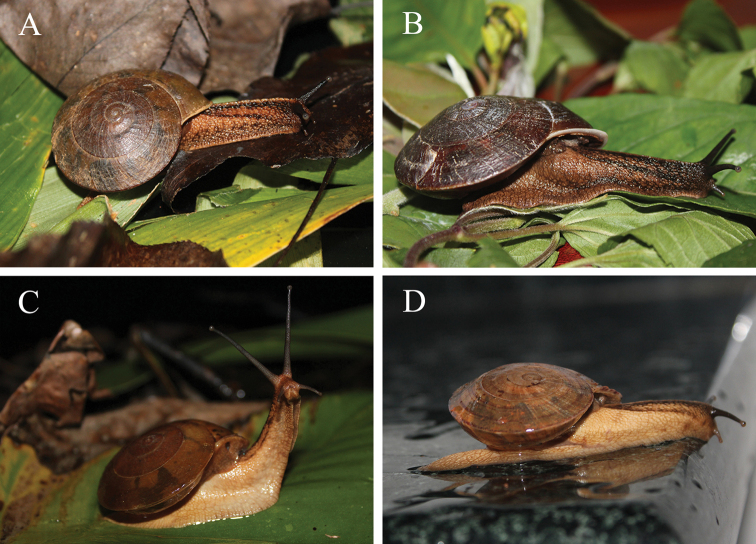
Ecological photographs of snails **A***Camaenafuningensis* sp. nov. (Laolida, Funing, Yunnan, China) **B***Camaenagaolongensis* sp. nov. (Dayao, Gaolong, Guangxi, China) **C***Camaenamaguanensis* sp. nov. (Huazhige, Maguan, Yunnan, China) **D***Camaenayulinensis* sp. nov. (Longquan cave, Yulin, Guangxi, China).

**Figure 6.**
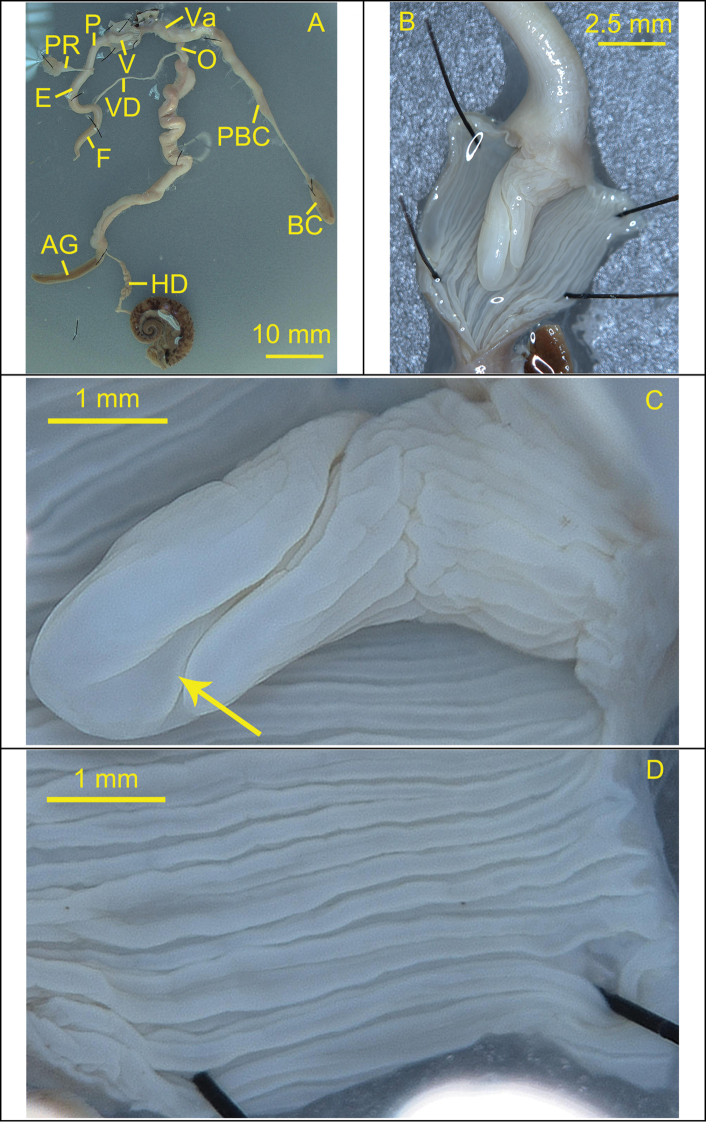
Reproductive system of the snail *Camaenafuningensis* sp. nov. (holotype, FJIQBC 19340, Laolida, Funing, Yunnan, China) **A** reproductive organ **B** penis **C** verge **D** inner penial wall. The arrow indicates opening position of the verge.

Figures 3A , 4 , 5A , 6 , Tables 3 , 4 , 5

###### Type material.

***Holotype*.** [FJIQBC 19340] Shell height 21.0 mm, shell width 41.0 mm, height of aperture 14.0 mm, width of aperture 18.7 mm, 22 October 2014, collected from the type locality.

***Paratype*.** [FJIQBC 19341–19343] 3 live specimens: 2 adults, 1 juvenile.

###### Type locality.

Laolida, Funing, Wenshan, Yunnan, China (23°31'48.88"N﻿, 105°32'59.70"E).

###### Etymology.

The name of the new species refers to the type locality.

###### Diagnosis.

***Shell*.** Shell dextral, large, thin, fragile and lucent, low, and flat conical. 4.5 whorls, the front whorls increasing slowly. Spire relatively low. Body whorl rapidly expanded. Shell light yellowish brown with clear growth lines and spiral bands on the surface. Apex quite blunt. Suture shallow. The protoconch surface smooth, and some short clear growth lines near the inner side of suture under 32 × stereomicroscope. Body whorl with carinate periphery, and a thin reddish brown band on the carina and several sparse bands below the carina. Aperture lunate, slightly descending. Peristome reflected, white, thin, sharp. Columellar lip reflected. Umbilicus reddish brown, large, only 1/5 covered. Inner lip attached to the body whorl, forming translucent callus.

***Soft body*.** Yellowish brown with irregular black lines and spots. Tentacles dark.

***Reproductive system*.** Bursa copulatrix oval and large with long and tapering pedunculus, expanded at the base. Flagellum long, tapering distally. Vas deferens short and thin. Epiphallus long, slightly thick. Penis retractor muscle medium length and slender, becoming wider at the end. Penis swollen and long, with longitudinal, slightly corrugated, strong and widely spaced pilasters internally. Verge ovate, opened terminally, and one clear crack on the verge surface extending from the terminal to the base.

###### Habitat.

The species was found on limestone.

###### Distribution.

Only known from the type locality.

###### Remarks.

*Camaenafuningensis* sp. nov. is characterized by a more oblate shape, lower spire, thin and fragile shell, and yellowish brown coloration, which are clearly different from the other dextral camaenids except *C.longsonensis* (Morlet, 1891), *C jinpingensis* Chen, Zhang & Li, 1990, and *C.vorvonga* (Bavay & Dautzenberg, 1900) (Chen et al. 1990; Schileyko 2011). The shells of the above four species are distinct from *C.funingensis* in the following ways:

(1) The umbilicus of *C.funingensis* sp. nov. is only 1/5 covered, while that of *C.longsonensis* is almost covered by reflected columellar lip leaving only a narrow slit, and that of *C.jinpingensis* is fully covered.

(2) *C.funingensis* sp. nov. has several reddish brown bands at the bottom of the body whorl in addition to those on the carina, while only one thin reddish brown band is present on the carinate periphery of *C.vorvonga*.

(3) For *C.funingensis* sp. nov., the verge is ovate and has one clear crack on the surface extending to the base, which makes it stand out other dextral camaenids.

*Camaenagaolongensis* sp. nov. is distinguishable from *C.funingensis* sp. nov. in having no spiral band. For *C.maguanensis* sp. nov., there is no band on the carinate periphery of the body whorl except for several below the carina. Moreover, the verge of *C.maguanensis* sp. nov. is small and circular. *Camaenayulinensis* sp. nov. differs to *C.funingensis* sp. nov. in having a conical verge and flesh-colored peristome.

*P*-distances of the COI gene between *C.funingensis* sp. nov. and the other camaenids are 0.068–0.200 (Table 3), and those between *C.funingensis* sp. nov. and *C.gaolongensis* sp. nov., *C.maguanensis* sp. nov. and *C.yulinensis* sp. nov. are 0.075, 0.068 and 0.094 respectively. All of these *P*-distances exceed the maximum intra-specific value 0.059 in the family Camaenidae. On the phylogenetic tree, these four new species are adjacent, hence it is reasonable to designate this as a new species.

##### Camaena gaolongensis

Taxon classificationAnimaliaCamaenidae

Zhou, Wang & Lin sp. nov.

DB60DEBD-A2E0-5365-B9C3-8E73A5D60C68

http://zoobank.org/1B657A19-59B9-46D2-B874-9DB7120730E9

**Figure 7.**
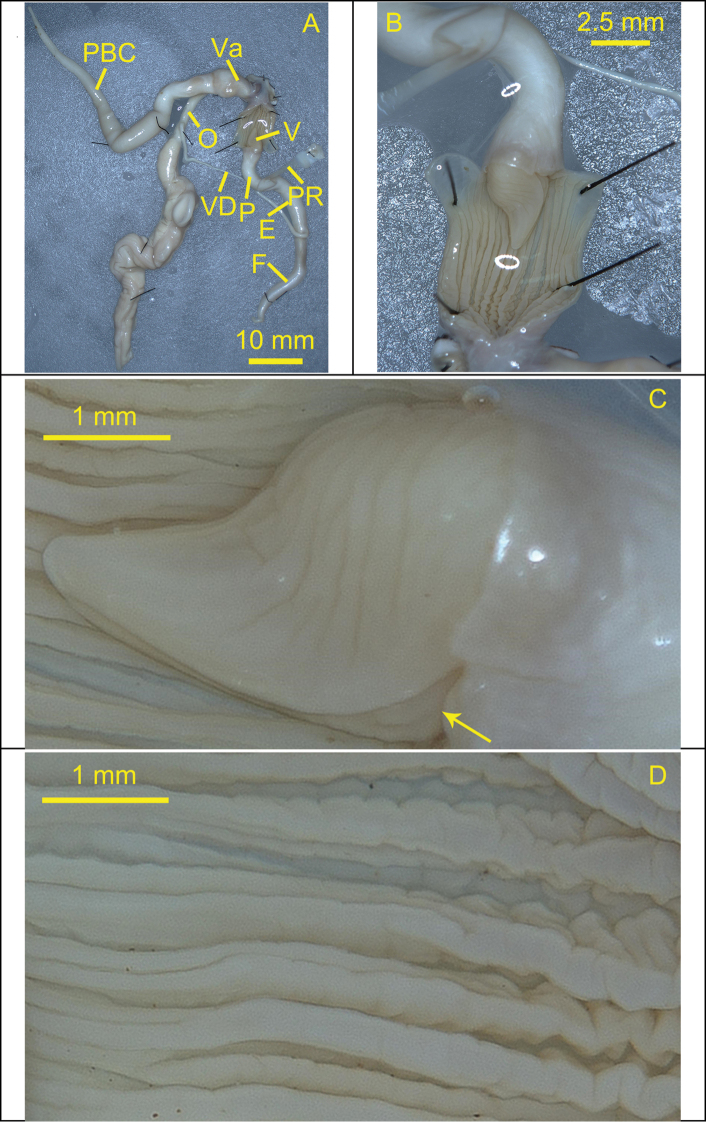
Reproductive system of the snail *Camaenagaolongensis* sp. nov. (holotype, FJIQBC 19353, Dayao, Gaolong, Guangxi, China) **A** reproductive organ **B** penis **C** verge **D** inner penial wall. The arrow indicates opening position of the verge.

Figures 3B , 4 , 5B , 7 , Tables 3 , 4 , 5

###### Type material.

***Holotype*.** [FJIQBC 19353] Shell height 23.8 mm, shell width 49.0 mm, height of aperture 14.0 mm, width of aperture 19.2 mm, 11 April 2015, collected from the type locality.

***Paratype*.** [FJIQBC 19354] 1 live juvenile, 20 October 2014; [FJIQBC 19355–19356] 2 live adults, 11 April 2015.

###### Type locality.

Dayao, Gaolong, Tianlin, Guangxi, China (24°11'52.33"N, 105°43'40.56"E).

###### Etymology.

The name of the new species refers to the type locality.

###### Diagnosis.

***Shell*.** Shell dextral, large, thick, strong, low, and flat conical. 4.5 whorls, the front whorls increasing slowly. Spire relatively low. Body whorl rapidly expanded. Shell dark brown with clear and dense growth lines on the surface. Apex quite blunt. Suture shallow. The protoconch surface smooth with scale marks, and some short growth lines clear near the outer side of suture under 32 × stereomicroscope. Body whorl with acute and carinate periphery, but no spiral band. Aperture U-shaped. Peristome reflected, white and thick. Columellar lip reflected. Umbilicus reddish brown, open, large, and only 2/5 covered. Inner lip attached to the body whorl, forming translucent callus.

***Soft body*.** Brown with irregularly black lines and spots. Tentacles dark.

***Reproductive system*.** Bursa copulatrix oval and medium sized with long pedunculus, expanded at the base, becoming thinner at the distal end. Flagellum long and smooth, tapering distally. Vas deferens long and thin. Epiphallus medium length and thick. Penis retractor muscle short, slender basally but wide and flat distally. Penis thick and medium length. Inner penial wall supporting longitudinal, stronger, and more widely spaced pilasters, smooth basally, curved distally. Verge irregularly conical, opened basally, extending from the base to the end, with several slanted wrinkles on the surface.

###### Habitat.

It is common in primary forest and loess areas, but it has not been found on the reclaimed lands outside the primary forest.

###### Distribution.

Only known from the type locality.

###### Remarks.

*Camaenagaolongensis* sp. nov. is clearly different from other dextral camaenids by its quite thick, low, flat, and dark brown conical shell resembling a flying saucer (Chen et al. 1990, Schileyko 2011). Additionally, the longitudinal pilasters on the inner penial wall are stronger and more widely spaced, as well as smooth at the base but curved at the end, which are also distinct from the other dissected *Camaena* snails (Ding et al. 2016, Ai et al.2016).

*P*-distances of the COI gene between *C.gaolongensis* sp. nov. and other dextral *Camaena* species are 0.075–0.203 (Table 3), and those between *C.gaolongensis* sp. nov., *C.maguanensis* sp. nov., and *C.yulinensis* sp. nov. are 0.085 and 0.104 respectively. Combining the topological structure of the phylogenetic tree, the new species *C.gaolongensis* sp. nov. is distinct from other dextral *Camaena* species.

##### Camaena maguanensis

Taxon classificationAnimaliaCamaenidae

Zhou, Wang & Hu sp. nov.

69AD598F-4236-5739-904B-4E8FC710E71B

http://zoobank.org/EC5431C5-CFB6-4309-80C1-0CF8F3C9BE0E

**Figure 8.**
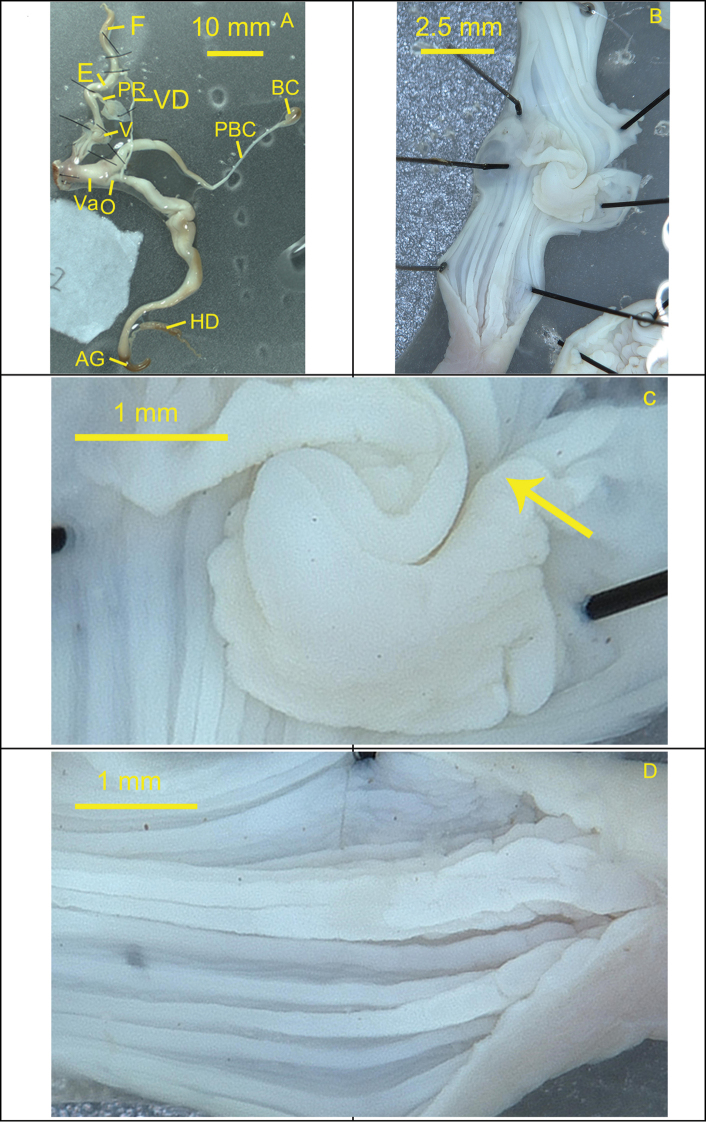
Reproductive system of the snail *Camaenamaguanensis* sp. nov. (FJIQBC 19405, Huazhige, Maguan, Yunnan, China) **A** reproductive organ **B** penis **C** verge **D** inner penial wall. The arrow indicates opening position of the verge.

Figures 3C , 4 , 5C , 8 , Tables 3 , 4 , 5

###### Type material.

***Holotype*.** [FJIQBC 19405] Shell height 19.2 mm, shell width 39.0 mm, height of aperture 12.0 mm, width of aperture 16.5 mm, 16 April 2015, collected from the type locality.

***Paratype*.** [FJIQBC 19406] 1 live adult; [FJIQBC 19407–19413] 7 empty shells: 5 adults, 2 juveniles.

###### Type locality.

Huazhige, Maguan, Wenshan, Yunnan, China (22°57'24.48"N, 104°21'12.96"E).

###### Etymology.

The name of the new species refers to the type locality.

###### Diagnosis.

***Shell*.** Shell dextral, large, thin, fragile, and glossy, low and flat conical. 4.5 whorls, the front whorls increasing slowly. Spire relatively low. Body whorl rapidly expanded. Shell yellowish with unclear growth lines and spiral bands on the surface. Apex quite blunt. Suture shallow. The protoconch surface smooth, some short growth lines visible near the two sides of suture under 32 × stereomicroscope. Last whorl with quite acute carina at periphery and a shallow groove-like depression above and below the carina. No band on the carina, but several reddish brown and sparse spire bands below the carina. Aperture crescent-shaped. Peristome reflected, white and thick. Columellar lip reflected. Umbilicus reddish brown, open, large and only 2/5 covered. Inner lip attached to the body whorl, forming translucent callus.

***Soft body*.** Light yellowish brown with black lines. Tentacles dark.

***Reproductive system*.** Bursa copulatrix oval, small, with quite long and tapering pedunculus. Flagellum long, tapering distally. Vas deferens long and thin. Epiphallus medium thickness and length. Penis retractor muscle very short and slender. Penis long with a short protrusion at the middle. Inner penial wall with longitudinal, slightly straight and smooth pilasters. Verge circular, somewhat small, opened basally, extending from the base to the end.

###### Habitat.

The species was found on limestone in Maguan county of Yunnan province, China.

###### Distribution.

Only known from the type locality.

###### Remarks.

*Camaenamaguanensis* sp. nov. is clearly different from other dextral camaenids with a lower conical shell. In particular, *C.maguanensis* sp. nov. has a large and open umbilicus, which distinguishes it from *C.longsonensis* and *C.jinpingensis*. Although the umbilicus of *C.maguanensis* sp. nov. is similar to that of *C.vorvonga*, some differences are obvious. For example, *C.maguanensis* sp. nov. has no spiral band on the carinate periphery of the body whorl but some spaced bands at the base. The shell of *C.maguanensis* sp. nov. is yellowish, but that of *C.gaolongensis* sp. nov. is dark brown. On the other hand, *C.maguanensis* sp. nov. has a circular and slightly smaller verge.

*P*-distances of the COI gene between this new species and the other dextral species are 0.068–0.198 (Table 3), and that between *C.maguanensis* sp. nov. and *C.yulinensis* sp. nov. is 0.108, also exceeding 0.059 (currently the maximum differentiation value (p-distance) of Camaenidae) (Criscione and Köhler 2014), and the topology of the phylogenetic tree also supports the new species.

**Table 4.** Adult shell dimensions (mm).

Species	*C.funingensis* sp. nov.	*C.gaolongensis* sp. nov.	*C.maguanensis* sp. nov.	*C.yulinensis* sp. nov.
Voucher	FJIQBC19340–19342	FJIQBC19353	FJIQBC19405–19411	FJIQBC19460–19466
		FJIQBC19355–19356		FJIQBC19468–19470
Sample size	3	3	7	10
SH	19.5–21.0	23.5–24.5	19.2–22.0	19.8–23.0
	(20.17±0.62)	(23.93±0.42)	(20.36±0.90)	(21.35±1.05)
SW	39.2–41.0	47.0–50.0	38.0–40.5	37.0–42.6
	(40.23±0.76)	(48.67±1.25)	(39.24±0.74)	(40.54±1.58)
SW/SH	1.95–2.03	2.00–2.06	1.84–2.03	1.84–1.96
	(2.00±0.03)	(2.03±0.02)	(1.93±0.06)	(1.90±0.03)
AH	13.4–14.0	13.8–14.2	12.0–13.1	13.0–14.6
	(13.63±0.26)	(14.00±0.16)	(12.64±0.34)	(13.76±0.48)
AW	18.0–18.7	19.0–19.4	16.5–18.1	17.5–21.6
	(18.30±0.29)	(19.20±0.16)	(17.22±0.56)	(19.11±1.46)
AW/AH	1.34–1.35	1.37–1.38	1.33–1.39	1.33–1.48
	(1.34±0.01)	(1.37±0.00)	(1.36±0.02)	(1.39±0.06)

##### Camaena yulinensis

Taxon classificationAnimaliaCamaenidae

Zhou, Wang & Hu sp. nov.

3E0CDA8E-7D14-53E3-8424-8FEF2D082342

http://zoobank.org/3038DBDB-A3B2-4364-B2D3-CB7E694EA8ED

**Figure 9.**
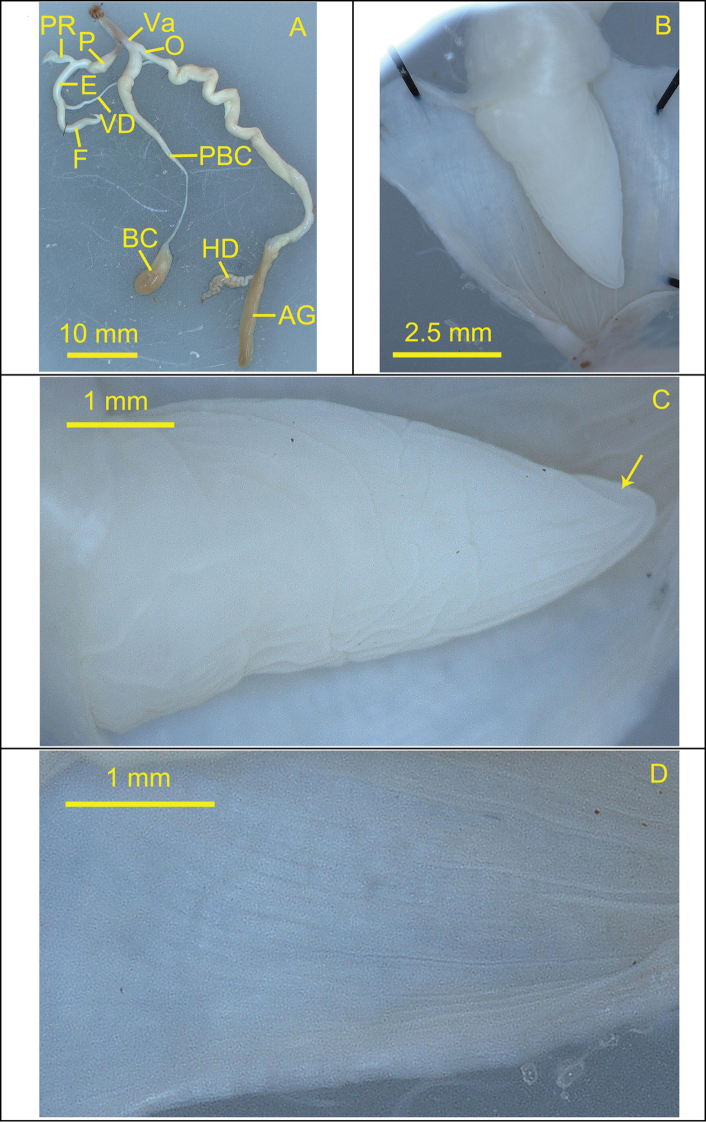
Reproductive system of the snail *Camaenayulinensis* sp. nov. (FJIQBC 19460, Longquan cave, Yulin, Guangxi, China) **A** reproductive organ **B** penis **C** verge **D** inner penial wall. The arrow indicates opening position of the verge.

Figures 3D , 4 , 5D , 9 , Tables 3 , 4 , 5

###### Type material.

***Holotype*.** [FJIQBC 19460] Shell height 21.0 mm, shell width 40.5 mm, height of aperture 13.5 mm, width of aperture 18.2 mm, 21 September 2014, collected from the type locality.

***Paratype*.** [FJIQBC 19461–19466] 6 specimens: 3 live adults, 3 empty adult shells, 4 November 2013; [FJIQBC 19468–19472] 5 specimens: 3 live adults, 2 empty juvenile shells, 21 September 2014.

###### Type locality.

Longquan cave, Yulin, Guangxi, China (22°36'41.24"N, 109°45'21.36"E).

###### Etymology.

The name of the new species refers to the type locality.

###### Diagnosis.

***Shell*.** Shell dextral, large, thin, fragile, and slightly lucent, low and flat conical. 4.5 whorls, the front whorls increasing slowly. Spire relatively low. Body whorl rapidly expanded. Shell light yellowish with clear and dense growth lines and spiral bands on the surface. Apex quite blunt. Suture shallow. The protoconch surface smooth for most individuals, but a few are rough. Growth lines clear near the outer side of suture under 32 × stereomicroscope. Last whorl with carinate periphery, a thin reddish brown spiral band on the carina, and many reddish brown spiral bands of different thickness on the upper and lower parts. Aperture lunate. Peristome reflected, flesh-colored, thin, sharp. Columellar lip reflected. Umbilicus reddish brown, open, large, and only 1/3 covered. Inner lip attached to body whorl, forming translucent callus.

***Soft body*.** Pale yellow with irregular black lines. Tentacles dark brown.

***Reproductive system*.** Bursa copulatrix oval, large, with long and tapering pedunculus. Flagellum long and slightly thick, tapering distally. Vas deferens short and thin. Epiphallus medium length and slightly thick. Penis retractor muscle short and wide. Penis short and swollen at distal 1/3, with longitudinal, thin, smooth pilasters internally. Verge conical, large, opened terminally, with some irregular wrinkles on the surface.

###### Habitat.

The species was found on limestone in Yulin city, Guangxi province.

###### Distribution.

Only known from the type locality.

**Table 5.** Diagnostic comparisons of morphological characters of the four new species.

Character	*C.funingensis* sp. nov.	*C.gaolongensis* sp. nov.	*C.maguanensis* sp. nov.	*C.yulinensis* sp.nov.
Shell thickness	thin	quite thick	thin	thin
Shell color	light yellowish brown	dark brown	yellowish	light yellowish
Periphery	carinate	acute and carinate	acute and carinate	carinate
Growth lines	clear	clear and dense	unclear	clear and dense
Umbilicus	only 1/5 covered	only 2/5 covered	only 2/5 covered	1/3 covered
Verge	ovate	short conic	circular and small	long conic
Verge opening	terminally, one clear crack on the surface extending from the end to the base	basally, one crack on the side surface extending from the base to the end	basally, one crack on the surface extending from the base to the end	terminally

###### Remarks.

*Camaenayulinensis* sp. nov. differs from *C.longsonensis* and *C.jinpingensis* in the key characteristic of large open umbilicus. This new species not only has spiral bands with different thickness on the body whorl but also has a flesh-colored peristome compared to *C.vorvonga*. The differences between this species and the other three new *Camaena* species herein have already been described above.

*P*-distances of the COI gene between *C.yulinensis* sp. nov. and the other dextral congeners ranges from 0.092 to 0.202 (Table 3) and the phylogenetic topology tree supports the establishment of this new species.

## Discussion

We describe four new species of dextral *Camaena* snails, namely *C.funingensis* sp. nov., *C.gaolongensis* sp. nov., *C.maguanensis* sp. nov. and *C.yulinensis* sp. nov., which are distinguished from their congeners by their shell morphologies, especially the low and flat shell shape, the large open umbilicus, the acute and carinate periphery of the body whorl, as well as features in their reproductive systems and molecular characteristics. Among the first three new species, the differences of shells and genitals are obvious. Although *C.funingensis* sp. nov. and *C.yulinensis* sp. nov. are similar in shell morphology except size, color and umbilicus, the former has an ovate and terminally opened verge and one clear crack on the surface extending from the end to the base, as well as strong and widely spaced penis pilasters, that distinguish it from *C.yulinensis* sp. nov. with a conical verge, thin penial inner pilasters and without crack on the surface (Figs 3, 5–9). Nonetheless, the two similar-shaped species are relatively distantly related genetically (Fig. 2).

Some scholars have considered genetic distance as one of the more important pieces of evidence used for identifying new species and revising species; for example, in the Asian camaenids *Luchuhadra* (Kameda et al. 2007) and *Satsuma* (Wu et al. 2008), the Australian camaenid *Kimberleytrachia* (Criscione and Köhler 2014), and *Camaena* (Ai et al. 2016; Ding et al. 2016). In the present study, the *p*-distances between *C.funingensis* sp. nov., *C.gaolongensis* sp. nov., *C.maguanensis* sp. nov., *C.yulinensis* sp. nov., and the other dextral *Camaena* was substantial: 0.068–0.200, 0.075–0.203, 0.068–0.198, and 0.092–0.202 respectively for the mitochondrial COI barcoding region (Table 3). These numbers exceed the intra-specific differentiation values (*p*-distances) of Camaenidae (for *Camaena*, minimum 0.00, maximum 0.018 in Ding et al. (2016), minimum 0.00, maximum 0.019 in Ai et al. (2016), for *Kimberleytrachia*, minimum 0.00, maximum 0.059, mean 0.026 in Criscione and Köhler (2014). Based on these considerations, inter-specific differentiation supports the recognition of the four new species.

In the phylogenetic analyses, *C.vorvonga* and *C.longsonensis*, which were placed in informal subgeneric group I, have a close relationship, while they are distant from *C.jinpingensis* that originally also belonged to group I. In the future, more species and sequences will be needed for a more robust analysis of camaenid phylogeny.

During our long-term field investigations, we observed that most *Camaena* species have a narrow distribution and a low population density, and only inhabit primary forests. An exception to this is *C.cicatricosa*, which is widespread and has high population densities (Ai et al. 2016; Ding et al. 2016). In recent years, with the development of the Chinese economy, areas of primary forest have been decreasing and the habitats of *Camaena* species are becoming increasingly restricted and threatened. Therefore, it is necessary to maximize forest protection, prevent deforestation, and prevent excessive tourist development to preserve the biodiversity of these terrestrial mollusks and other animals and plants.

## Supplementary Material

XML Treatment for Camaena

XML Treatment for Camaena funingensis

XML Treatment for Camaena gaolongensis

XML Treatment for Camaena maguanensis

XML Treatment for Camaena yulinensis
